# A task-sharing intervention for prepartum common mental disorders: Feasibility, acceptability and responses in a South African sample

**DOI:** 10.4102/phcfm.v12i1.2378

**Published:** 2020-10-01

**Authors:** Maxine Spedding, Dan J. Stein, Tracey Naledi, Bronwyn Myers, Pim Cuijpers, Katherine R. Sorsdahl

**Affiliations:** 1 Department of Psychology, Faculty of Humanities, University of Cape Town, Cape Town, South Africa; 2 Department of Psychiatry, Faculty of Health Sciences, University of Cape Town, Cape Town, South Africa; 3 Department of Public Health, Faculty of Health Sciences, University of Cape Town, Cape Town, South Africa; 4 Alcohol, Tobacco and Other Drug Research Unit, South African Medical Research Council, Cape Town, South Africa; 5 Department of Clinical, Neuro and Developmental Psychology, Faculty of Behavioural and Movement Sciences, Vrije Universiteit, Amsterdam, the Netherlands

**Keywords:** primary healthcare, task-sharing, mental health, acceptability, peripartum care

## Abstract

**Background:**

Peripartum common mental disorders (CMD) are highly prevalent in low- and middle-income countries (LMIC) such as South Africa. With limited public mental health resources, task sharing approaches to treatment are showing promise. However, little is known about the feasibility and acceptability of, as well as responses associated with problem-solving therapy (PST) for the treatment of prepartum CMD symptoms in South African public health settings.

**Aim:**

To investigate participants’ preliminary responses to a task sharing PST intervention, and to evaluate the feasibility and acceptability of the intervention.

**Setting:**

A Midwife and Obstetrics Unit attached to a Community Health Centre in a Western Cape district.

**Methods:**

Using mixed methods, 38 participants’ responses to a PST intervention, and their perceptions of its feasibility and acceptability, were explored. Primary outcomes included psychological distress (Self Reporting Questionnaire; SRQ-20) and depression symptoms (Edinborough Postnatal Depression Scale; EPDS). Semi-structured interviews were conducted three after the last session. Six stakeholders were also interviewed.

**Results:**

Significant reductions were seen on EPDS (Cohen’s *d* = 0.61; Hedges *g* = 0.60) and SRQ-20 (Cohen’s *d* = 0.68; Hedges *g* = 0.67) scores. The intervention’s acceptability lay in the opportunity for confidential disclosure of problems; and in relieving staff of the burden of managing of patients’ distress. Barriers included lack of transport and work commitments.

**Conclusion:**

Results support task sharing PST to Registered Counsellors to treat antenatal CMDs in perinatal primary health care settings. Research is needed on how such programmes might be integrated into public health settings, incorporating other non-specialists.

## Introduction

Perinatal common mental disorders (CMDs), such as depression and anxiety, are highly prevalent in low-and middle-income countries (LMICs)^1^ and are associated with a range of adverse outcomes for mothers and infants.^2,3^ Yet, in LMICs, up to 90% of people who could benefit from mental health treatment do not receive care.^4^ In South Africa, three out of four people with CMDs do not receive treatment.^5^ As a means to address this treatment gap, task-sharing mental health interventions to non-specialist health workers (NSHW) has garnered increasing attention.^6^ By extension, there is a growing body of evidence showing that task-sharing interventions to treat perinatal CMDs are feasible to deliver, acceptable and also effective.^7^

Several systematic reviews have investigated the effectiveness of task-sharing mental health interventions in LMICs.^8,9^ One systematic review of 13 studies found that task-sharing interventions improved maternal mental health, which had a positive impact on infant development and health.^7^ Correspondingly, improving mothers’ ability to respond to their infants’ needs also improved maternal mood.^7^ Similarly, another review pooled data from 10 trials of psychosocial interventions delivered by NSHW in community settings and antenatal units aimed at reducing perinatal CMDs.^10^ They found that, compared to usual care, interventions led to an overall reduction in CMD symptoms when using continuous data for symptomology.^11^

As one of the World Health Organization’s (WHO) Mental health Gap Action Programme (mhGAP)-recommended treatments,^12^ problem-solving therapy (PST) has found significant support as an easily adaptable, user-friendly and task-sharable psychotherapy.^13,14,15,16^ Evidence suggests that it is an effective treatment for several CMDs, including mood,^17^ anxiety,^18^ psychological distress^19^ and substance use disorders^20^ in a broad range of sociocultural settings.^21^ In LMICs, the evidence for PST is growing. In Zimbabwe, Chibanda et al.^13^ found that three to six sessions of PST delivered by lay workers significantly reduced CMD symptoms in a sample of 320 adults. Another study showed that levels of psychological distress were significantly lowered using a PST intervention in a South African sample of 103 participants.^19^ Also in South Africa, a trial of blended motivation interviewing and PST amongst 335 patients attending emergency care reported significant reductions in substance use and depression 3 months post-enrolment.^22^ Notably, Chibanda et al.^23^ found that, at 6 weeks post-intervention, depression scores of a group of women receiving a PST intervention were significantly lower than those who received antidepressant medication.

Given the comparatively recent recognition of the burden associated with maternal mental illness in LMICs,^1^ there are extensive gaps in our knowledge. Firstly, limited research has been conducted on the efficacy of task-sharing evidence-based interventions that are integrated into antenatal primary healthcare. Whilst there is evidence to support the use of PST to treat depression,^14^ the evidence for its application to peripartum CMDs is limited internationally and absent in South Africa. Secondly, little is known about the feasibility and acceptability of mental health interventions that are integrated into antenatal healthcare services, for both participants and stakeholders. As such, this article aims to describe (1) women’s preliminary responses to the PST intervention, (2) to explore women’s perceptions of the intervention’s feasibility and acceptability, and (3) to explore healthcare providers’ perceptions of barriers to and facilitators of integrating a PST intervention into midwife and obstetrics unit (MOU) services.

## Methods

### Setting

Data were collected at a MOU that serves a large district in the Western Cape province of South Africa, with a primarily low-income population of more than 300 000 people.^24^ Midwife and obstetrics units fall under the governance of the Western Cape’s Department of Health and provide a range of perinatal services at primary care level, including antenatal check-ups, deliveries by midwives and postnatal care for mothers and infants. They are usually attached to a primary healthcare in areas that were classified as ‘black African’ or ‘coloured’^i^ under the apartheid regime, serving previously disadvantaged communities. Eighty-four per cent of the South African population are dependent on government-funded health services.^25^

### Design and procedures

We employed a mixed-methods design comprising two phases. In phase 1, quantitative data were collected to measure participants’ preliminary responses to the intervention, whilst qualitative data were collected to explore women’s perceptions of its feasibility and acceptability. This phase addressed the study’s first two aims. In phase 2, qualitative methods were again used to collect data concerning the MOU personnel’s (stakeholders) perceptions of the barriers to and facilitators of intervention delivery. This phase addressed the study’s third aim. Given the small sample sizes in both phases, qualitative methods for examining feasibility and acceptability were deemed most appropriate.

#### Phase 1: Participant responses to and perceptions of feasibility and acceptability

**FIGURE 1:**
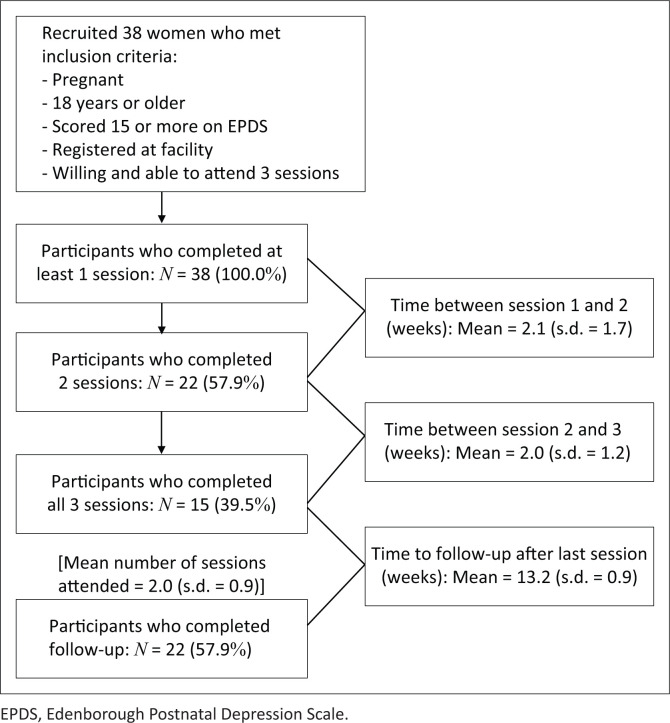
Recruitment procedures and intervention process.

**Participants:** Over a period of 1 year, a purposive sampling was used to recruit 38 pregnant women to participate in the study (see Figure 1). To be eligible to participate in the study, women had to be pregnant, at least 18 years old, must have registered for care at the facility and must have scored 15 or more on the Edenborough Postnatal Depression Scale (EPDS) during the standard intake interview conducted by the intake nurse. All eligible women were given a referral to the registered counsellor. Those who accepted the referral were asked to provide written informed consent to participate in the study.

**Procedure:** Following the recruitment all participants completed a baseline assessment. Immediately following the assessment, they received the first of three PST sessions. The second and third sessions were scheduled about a week apart from each other. Patients deemed at risk for suicide, or with signs and symptoms of other serious pathology, were referred for specialist care. Three months after their last PST session, participants were asked to return to the MOU where the baseline questionnaire was re-administered by two research assistants. Short qualitative interviews explored the acceptability and perceived benefits of the intervention and asked for suggestions regarding the content and procedural improvements. All interviews were audio-recorded and transcribed. At the baseline and follow-up assessments, the participants were given a grocery store voucher as a token of thanks for their time.

#### Intervention

The PST intervention used in this study was adapted from Sorsdahl et al.^20^ Focus groups were conducted with 12 women who met the inclusion criteria to gather feedback on the intervention and how it should be adapted for pregnant women. Adaptations were primarily target population-related and included the addition of information about the experience of pregnancy. An outline of the session content and procedures is presented in Table 1. All sessions incorporated worksheets intended for use during the session, as well as homework assignments.

**TABLE 1:** Description of the problem-solving therapy sessions’ content and procedures.

Session 1	Session 2	Session 3
In addition to orienting the participant to the PST model, this session involved helping her identify what is most important to her life. Problems and worries would then be listed and categorised in one of three groups: problems that are not important (group A), problems that are important but unsolvable (group B) and problems that are important and solvable (group C). A problem from the third group would then be selected and together the registered counsellor and participant would develop a step-by-step plan to solve the problem.	The participant would be reminded of the PST model and a list of adaptive coping strategies would be discussed. A problem from the ‘not important’ category would then be selected and the ways in which coping strategies might be applied to these would be discussed. In addition, a problem from the third group would be selected and together the registered counsellor and the participant would develop a step-by-step plan to solve the problem.	Again, the participant would be reminded of the model and then more coping strategies would be discussed. Thereafter, a problem from the ‘important but unsolvable’ category would be selected from the list made in the first session, and ways of coping with this problem would then be discussed. Again, a problem from the third group would also be selected and together registered counsellor and the participant would develop a step-by-step plan to solve the problem.

Intake nurses were trained to use the EPDS, incorporate it into standard assessment procedures and make referrals to the registered counsellor. In addition to her formal Bachelor of Psychology degree training, the registered counsellor also received a 3-day training course in maternal mental health and 18 h of training in the PST model and manual. She also received at least 1 h of clinical supervision per week from the first author, a registered clinical psychologist. Procedural matters, case management and fidelity to the therapeutic protocol were addressed in supervision. A random review of recorded sessions did not reveal any protocol drift.

### Measures

The primary outcome of this study was psychological distress. The secondary outcomes included perinatal depression, functional impairment, substance use involvement, perceived stress and perceived social support.

*Psychological distress*: The Self-Reporting Questionnaire (SRQ-20)^26^ is a 20-item screening tool designed to screen for symptoms associated with a range of CMDs. A cut-off value of ≥ 8 was used to determine caseness, producing the binary categories of ‘high’ (≥ 8) and ‘low’ (≤ 7).^27^ The SRQ-20 has satisfactory sensitivity and specificity.^28^

*Symptoms associated with perinatal CMDs*: The EPDS^29^ is a 10-item scale that screens for symptoms of perinatal CMDs in the last 7 days. It is one of the most validated tools in LMICs.^30^ A cut-off value of ≥ 15 was used to determine caseness, yielding binary categories of ‘high’ (≥ 15) and ‘low’ (≤ 14).^31^

*Functional impairment*: The Sheehan Disability Scale (SDS)^32^ was used to assess functional impairment in three inter-related domains: work/school, social and family life. On a scale of 0 (being ‘not at all’) to 10 ( being ‘extremely’), participants were asked to rate the degree to which symptoms had disrupted their lives in these domains.

*Substance use involvement*: The Alcohol, Smoking and Substance Involvement Screening Test (ASSIST)^33^ was used to investigate self-reported substance use. Substance involvement scores are generated for each substance used in the 3 months prior to the interview. Scores of ≤ 3 (10 for alcohol) indicate a low risk for substance-related health problems, whilst scores between 4 and 26 (11–26 for alcohol) reflect a moderate risk and that ≥ 27 reflect a severe risk.^33^

*Perceived stress*: The Perceived Stress Scale (PSS)^34^ asks participants to respond on a scale of 0 (‘never’) to 4 (‘very often’) to a series of 10 questions examining the levels of perceived stress.

*Perceived support*: The Multidimensional Scale of Perceived Social Support (MSPSS)^35^ is a 12-item scale that asks participants about their current perceptions and experiences of being supported or assisted by family members, significant others and friends.

#### Phase 2: Stakeholder perceptions of the intervention’s feasibility and acceptability

**Participants:** A purposive sampling was used to recruit participants, as all stakeholders who were identified as being the most directly involved in or impacted upon by the project were invited and agreed to participate. They comprised three staff members who were most involved in the screening of participants at their first antenatal visits, the primary liaison person and acting head of the MOU, the Community Health Centre’s social worker and the registered counsellor who delivered the intervention (see Table 2).

**TABLE 2:** Stakeholders’ roles in the project.

Title/designation	Role in the project
Health worker	Screening and referral
Senior nursing assistant	Screening and referral
Midwife, nursing sister	Screening and referral
Midwife, nursing sister, acting head of MOU	Referral source, primary liaison
Social worker	Referral source and resource
Registered counsellor	Collected baseline data and delivered PST intervention

MOU, Midwife and Obstetrics Unit; PST, problem-solving therapy.

**Procedure:** Face-to-face interviews were arranged with stakeholders, who provided informed consent to participate under conditions of anonymity and voluntary participation. As the registered counsellor was under the clinical supervision of the first author at the time, she was interviewed by a clinical psychologist independent of the study. This was done to avoid any conflict of interest, as well as to minimise interviewer and response bias. A semi-structured interview schedule was used to guide the interviews with stakeholders. It included questions regarding stakeholders’ perceptions of the intervention’s utility to the service, as well as the impact of intervention on their day-to-day duties. All interviews were audio-recorded and transcribed. Once transcribed, the audio recordings were destroyed and all identifying information was removed from the transcribed material, which was kept on a password-protected computer.

### Data analysis for phases 1 and 2

Quantitative data were analysed using SPSS version 23.0. Participants’ socio-demographic data were analysed with descriptive statistics. We used both paired *t*-test and the Wilcoxon’s signed-rank test to assess the initial effect of the intervention on the primary and secondary outcome variables. The last observation carried forward method was used to impute missing data. We reported effect sizes using Cohen’s *d* and Hedges’ *g* for small samples. Qualitative data were analysed in NVivo 11 using the framework method.^35^ This approach to thematic analysis involves a series of stages that include familiarisation with the material, coding the transcripts, developing an analytical framework, applying the analytical framework by indexing, charting data into the framework matrix and interpreting the data.

### Ethical consideration

Ethical approval to conduct the study was obtained from the Faculty of Health Sciences Research Ethics Committee (HREC) at the University of Cape Town. Permission to collect data at the midwife and obstetrics unit was also obtained from the Western Cape Department of Health as well as the facility management. Written informed consent was obtained from all the participants. In order to protect the identities of the stakeholders, identification codes were omitted from quotes included in this article.

## Results

### Women’s preliminary responses to the problem-solving therapy intervention

Of the 38 women who participated in the study (see Table 3 for a description of socio-demographic characteristics of the participants), 15 (39.5%) attended all three sessions, 9 (23.7%) attended two sessions and 14 (36.8%) attended one session. The average number of sessions attended was 2.03 (standard deviation [s.d.] = 0.89). The mean number of weeks that elapsed between the first and second sessions was 2.12 (s.d. = 1.68) and that between the second and third sessions was 2.00 (s.d. = 1.16). Preliminary response data for 22 (57.8%) participants were obtained (see Figure 1). Sixteen participants were lost to follow-up because of withdrawal from the study, relocation to another area, a change in contact number or scheduling conflicts. Of those who participated in the follow-up interview, 10 (45.5%) completed all three sessions.

**TABLE 3:** The demographic characteristics of the problem-solving therapy intervention sample (*N* = 38).

Variable	Total sample (*N* = 38)	% of sample
**Age**		
18–24 years	11	28.9
≥ 25 years	27	71.1
**Relationship status**		
Partnered	21	55.3
Unpartnered	17	44.7
**Highest level of education completed**		
Primary school	24	63.2
High school	11	28.9
Tertiary qualification	3	7.9
**Race**		
Black African	8	21.1
Coloured†	30	78.9
**Religion**		
Islam	8	21.1
Christianity	30	78.9
**Main languages spoken at home**		
English	14	36.8
Afrikaans	16	42.1
isiXhosa	3	7.9
Other indigenous South African languages	3	7.9
Other languages	2	5.3
**Employment status**		
Unemployed	17	44.7
Unemployed by choice (student, homemaker)	8	21.1
Employed (full-time or part-time)	13	34.2
**Social assistance**		
None received	17	44.7
Childcare grant recipient	21	55.3
**Own average monthly income**		
< R1000/month (< ±US$74)	22	57.9
R1000 – R5000/month (±US$74 – US$370)	13	34.2
R5000 – R10000/month (±US$370 – US$740)	3	7.9

† , The authors are cognizant of the deeply and historically problematic use of this classification. However, given South Africa’s troubled socio-political history, the use of these markers allows for the monitoring of improvements in health and socio-economic disparities that originated within such a classification system.

Addressing the study’s first aim, which sought to investigate women’s preliminary responses to the intervention, several significant gains were seen on both primary and secondary outcome measures, as reflected in Table 4. Preliminary responses to the primary outcomes were positive, with significantly decreased EPDS scores (Cohen’s *d* = 0.61; Hedges’ *g* = 0.60) and SRQ-20 scores (Cohen’s *d* = 0.68; Hedges’ *g* = 0.67). Correspondingly, impairment to functioning was also reduced, with all three SDS reflecting less disruption to work (Cohen’s *d* = 0.42; Hedges’ *g* = 0.41), social life (Cohen’s *d* = 0.71; Hedges’ *g* = 0.69) and family and home responsibilities (Cohen’s *d* = 0.43; Hedges’ *g* = 0.42). Perceived Stress Scale scores were also significantly reduced (Cohen’s *d* = 0.63; Hedges’ *g* = 0.62). No other significant changes were observed.

**TABLE 4:** Participant pre-post differences in outcome measures imputing for missing data (*N* = 38).

Outcomes	Pre-intervention		Post-intervention		Comparison					
	Mean	s.d.	Mean	s.d.	Mean diff	s.e.	Correlation	Hedges’ *g*	Cohen’s *d*	*p*
**CMD symptoms (EPDS)**	19.4	3.6	16.2	6.3	3.2	1.1	0.222	0.60	0.61	< 0.01
**Psychological distress (SRQ20)**	14.9	3.6	11.3	6.1	6.3	1.2	0.484	0.67	0.68	< 0.01
**Disruption in functioning (Sheehan)**										
Work	5.9	3.2	4.5	3.5	2.5	0.9	0.44	0.41	0.42	0.02
Social life	7.2	3.1	4.6	4.1	6.8	0.5	0.386	0.69	0.71	< 0.01
Family life	6.5	3.5	4.9	3.9	5.6	1.0	0.456	0.42	0.43	0.01
**Perceived Stress (PSS)**	30.68	5.5	26.0	8.7	9.1	2.0	0.306	0.62	0.63	< 0.01
**Substance abuse (ASSIST)**										
Tobacco involvement score	14.0	14.2	14.5	13.2	0.7	1.6	0.912	0.04	0.04	0.66
Alcohol involvement score	6.7	9.7	6.9	10.8	−0.3	2.0	0.763	0.02	0.02	0.87
**Social support (MPSS)**										
Significant other	21.3	6.8	22.3	6.4	−1.8	1.1	0.836	0.15	0.15	0.11
Family	15.5	8.1	16.1	8.6	−1.1	1.9	0.709	0.07	0.07	0.56
Friends	15.9	8.3	16.8	8.1	−1.7	2.2	0.594	0.11	0.11	0.47
Overall social support	52.7	17.7	55.2	19.2	−4.6	3.5	0.789	0.13	0.13	0.21

CMD, common mental disorders; s.d., standard deviation; s.e., standard error.

### Feasibility and acceptability of the intervention^ii^

Data from interviews with women who participated in the intervention (participants) and staff members involved in the delivery of the project (stakeholders) highlighted several emergent themes, addressing the study’s second and third aims regarding the intervention’s feasibility and acceptability.

#### Perceptions of the intervention’s acceptability and usefulness

Most of the participants felt that they derived some benefit from the intervention, with nearly all participants reporting that they would recommend such a programme to friends. The opportunity to hear another perspective, to talk about past experiences or to have time for themselves was critical. The opportunity to confide in a non-judgmental person, who was not previously known, was deemed particularly helpful:

‘I found out I was pregnant. I didn’t want the baby, all of that and – it was so painful for me but – really after talking to her it – just to speak to someone that’s not family, man, someone you don’t know, really helped, it’s almost like it’s just a burden off your shoulder. Since the first session we had, I could see a light again and I could actually feel that this is my baby …’ (Participant #26, aged 28)

From these descriptions, PST-specific factors of the intervention may be less important to some participants than having the registered counsellor’s impartial and empathic ear. Other participants valued the problem-solving approach itself. Some participants expressed appreciation for its pragmatism, whilst others referred directly to the problem-solving aspects of the intervention as useful, such as the development of better coping mechanisms:

‘I learned to control the problems I have and how to solve it and what to do and so on. That is how it helped me.’ (Participant #24, aged 23)

‘How she taught me how to sit and think and not allow my thoughts to run through my mind but to allow it to let it stop and so on. Yes, think more about the positive things.’ (Participant #24, aged 23)

Reports on improved self-efficacy that in turn led to more positive feelings about themselves, such as ‘being stronger’ and feeling like ‘a better person’, were described. For some participants, these benefits were linked to improved relationships, whilst others found that they were more able to seek out social experiences than before:

‘… [*T*]he sessions really helped – with my relationship; things at home. I found I had solution after. I was a troubled person and to think low of myself. Now I can open conversations with people which I couldn’t do before.’ (Participant #13, aged 32)

A few participants spoke about using what they had learnt from the intervention to help others, going so far as to make copies of the booklet for friends. One participant even arranged to meet with her friends at the time that her weekly appointment with the registered counsellor had been due, to teach them the PST techniques.

On the other hand, two participants reported that they did not find the intervention helpful at all (one of them still attended all three sessions). Whilst both seemed to suggest that their objections were related to the registered counsellor’s style, it is possible that the PST model was a poor fit for them, as evident from the following comment: ‘there wasn’t really space for me to talk, we were just reading out of the booklet’. Despite one participant’s appreciation for the ways in which the model helped her better manage distressing thoughts, she also made an important observation about the limits of counselling interventions for someone who lives in poverty. In this instance, this participant highlighted the tension between the need to think about how to find money and the anxiety and stress that these thoughts generated for her:

‘I can think because I’ve got children, you see, I’ve got no job, I must make something for my children, but everything it’s use the money, you must have the money, you just think and keep thinking “where must I get the money? Where must I do that?” But the only thing is to stop thinking. I can’t stop thinking. I must think.’ (Participant #18, aged 29)

From the stakeholders’ perspective, most seemed to feel that the programme lightened their own workloads, as it gave them a resource to refer distressed patients to, instead of having to manage the patients themselves. Several stakeholders described how interactions with distressed patients could be burdensome and stressful for the staff, in that containing the patient took time and energy from their own limited resources, as highlighted in the following extract:

‘And I’m just asking [*the patient*] “so why are you smoking such a lot!” … Noooo, but then I end up having to hear about her being abused as a child and her husband is hitting her. And one question led to all of that … Sometimes you just don’t ask … Sometimes you just say, “I just want to get through the day, I’m not going to ask”.’ (Stakeholder #1)

All the stakeholders talked about the ways in which having a counsellor at the MOU relieved some of the demands that distressed patients represented. This seemed to be the most significant and meaningful contribution that the programme made to staff:

‘I think how it impacted on my work is that a lot of the clients that was actually screened to be seen by [*the Registered Counsellor*] eventually didn’t come to me because if she wasn’t there I’m sure that a lot of those cases would have come to me, so it impacted on my caseload going down.’ (Stakeholder #2)

#### Perceived barriers and challenges

Participants who had missed appointments with the registered counsellor or had prematurely terminated their participation provided several reasons for doing so. Although a few participants highlighted how stigma associated with attending counselling sessions at the MOU might be a barrier, such as ‘people will think something is wrong with you, structural barriers, mainly related to financial constraints and lack of transport, were the most commonly cited. Childcare and work commitments also presented obstacles to attending sessions:

‘Okay all her available times, then I was busy – it was either work or I had to be by my child’s school.’ (Participant #24, aged 23)

The stakeholders highlighted several challenges associated with the programme, many of which appeared to be related to an overburdened system. As referrals increased, the registered counsellor had less time to immediately see all patients referred to her and in some instances she would need to arrange an appointment on another day instead. When asked about what she thought was problematic about the programme, one stakeholder had this to say:

‘The amount of patients that was referred … Because I don’t think [*the Registered Counsellor*] could keep up with all the patients. And then she used to say she can’t see somebody now, she must get a date or whatever, then the staff just stopped referring.’ (Stakeholder #1)

The role that the programme played in relieving the staff members seemed to be echoed in this way and perhaps highlighted a sense of inadequacy or anxiety about having the capacity to manage a distressed patient. One stakeholder alluded to this:

‘But even those patients really need help so if – I mean [*the Registered Counsellor*] is not there on that particular day or maybe … or maybe or if [*the Registered Counsellor*] is fully-booked, so she has to give the other days … and you send the patient home without – being helped … because you can talk to the patient but you feel that it’s not enough, maybe it’s not enough. Of course I understand for the patient because there is somebody there that they talk [*to*], they [*are*] going to feel much better but for you that you’ve been talking to the patient you think, I don’t think I gave enough.’ (Stakeholder #4)

Every stakeholder made reference to the overburdened and understaffed state of the system and the consequent demands placed on staff members. Interestingly, whilst the value of the programme for the staff appeared to be in the relief it offered from the demands of distressed patients, feeling overburdened may have, in and of itself, represented a barrier to the acceptability of the programme. In this vein, two stakeholders expressed frustration at their colleagues for their unwillingness to participate in projects in general: ‘I think people work in little squares and they’re just concerned about what happens in their little square.*’* For one of the stakeholders, the reticence to participate was observed in colleagues, whilst frustration was understandable and explained by staff being overburdened and overworked:

‘I know, it also has to do with the lack of staff and the amount of clients that need to be seen. So I also understand that perhaps people feel overwhelmed and so [*speaking as if an affected staff member*] “I don’t really want to become too interested in something else because even if I want to do that I must still see to the fifty that’s waiting for me”.’ (Stakeholder #2)

The registered counsellor also reported that the intake nurses felt that the screening and referral process was burdensome:

‘Those that were involved – let’s just say the intake nurses found it to be a “las” (burden) – it’s extra work for them, [*as if quoting the staff*] “I’ve got to refer, I’ve now got to give you updates on my numbers, now you want to give them a sticker” – you know if we didn’t have stickers they had to fill in the forms for me by hand, so needed dates of birth, surnames, contact details.’ (Stakeholder #3)

The lack of space at the MOU was another obstacle to the acceptability of the programme, as stated by one stakeholder: ‘The only thing here is the space thing you understand?’ However, one stakeholder felt that the lack of space was sometimes used as a reason to prevent new programmes from being adopted, as new programmes often represent additional work. In this way, physical space may well have represented staff members’ capacity – in terms of time and energy – to accommodate the additional duties that programmes often bring with them:

‘It’s difficult because they agree to a lot of things and then when it needs to happen, there’s no space available, people don’t want to share their space.’ (Stakeholder #2)

#### Recommendations for improvement of the intervention

Given that a majority of participants stated that the intervention was acceptable the way that it was, few recommendations for improvements were made. Of those who provided recommendations, many stated that group sessions would be beneficial in providing support and that the intervention should be made available at other locations. This recommendation was to address the practical difficulties in accessing the clinic, or to protect participants from the stigma associated with receiving mental health services:

‘Ja [*yes*] house-visiting and stuff like that, that will help, for me because I am staying very far from the clinic. Maybe have it at other clinics.’ (Participant #15, aged 38)

Several participants stated that the number of sessions was inadequate and that more or longer sessions would improve the intervention:

‘Longer sessions because you just deal with this and now you get to, not a breaking point but you know, you get to a point where you think, where you feel there’s still a lot for you to resolve but the sessions is too *short*.’ (Participant #20, aged 31)

Despite some ambivalence, all stakeholders stated that having a counsellor at the MOU was essential. Stakeholders stated that they needed someone who would attend specifically to patients’ mental health, worrying that in the meantime, as the programme has terminated, staff might not detect problems:

‘We need our own counsellor. We are not picking up depression, we are not picking up postnatal depression because we’re not looking for it. As it is now there is a lot of people slipping through our hands that need help … And we are [*only*] focusing – mommy, stomach, baby – we [*are*] not focusing [*on*] mental health.’ (Stakeholder #6)

Three stakeholders reported that increasing both patient and staff awareness about mental health and counselling would improve the service and retention rates:

‘I think that if we can get to a point where we actually get people to understand that there’s more to wellness than just physical health um – we would have done a lot – maybe we can do a lot better then.’ (Stakeholder #2)

Two stakeholders stated that stigma associated with mental illness needed to be addressed in order to improve the programme. Both felt that being seen by other patients to use the service made the women feel self-conscious and therefore less inclined to take up counselling:

‘Maybe they don’t want to be seen – I think – when people don’t want to be seen – maybe they thought “this one knows me and they know I’m coming for this and I’m coming for that” – see them in a certain time or maybe give them appointments to come.’ (Stakeholder #4)

Despite the concern about space, one solution offered by several stakeholders to ensure that all patients are seen on the day was to have more counsellors available so that a walk-in service could be made possible. However, one stakeholder stated that a walk-in service would mean that counselling is treated as a ‘crisis service’. She opined that this would send the wrong message to women about taking care of their mental health:

‘I don’t know how effective that is also um – because it also creates the wrong perception with the client in terms of intervention and what can be done – and again in my opinion I think it would be better to say to people you know, mental health is a thing that you should pay attention to continuously and not only when you are in crisis or when there’s a problem.’ (Stakeholder #2)

## Discussion

This is the first study in South Africa to investigate the feasibility, acceptability and preliminary responses to an adapted PST intervention for psychologically distressed pregnant women. Quantitative data provide initial support for the potential benefits of the intervention for reducing symptoms of psychological distress and improving functioning. In line with findings from other studies,^13,19,23^ this study’s results showed reduced symptoms of CMDs and psychological distress. Improved functioning was seen on the work, social and family/home dimensions of the SDS. Qualitative data supported the feasibility and acceptability of the intervention, with participants reporting reductions in distress and improvements in social functioning, whilst stakeholders were generally positive, reporting some relief from having to manage patients’ psychological distress.

Retention rates of almost 40% for the full intervention, whilst not high, appear to be in line with prepartum mental health interventions conducted in other settings.^8,36^ One systematic review found that low-income women are more likely to discontinue therapeutic treatments prematurely, mainly for financial reasons.^37^ Indeed, a lack of transport or money and work commitments were reported as barriers by the participants of this study, as has been found in other studies.^37,38,39^ The implications for policy then are that simply increasing the number of available services or human resources for mental health is not adequate. Addressing these barriers might include developing after-hours services, providing transport coupons or delivering interventions at patients’ homes, when appropriate and possible. As many participants could not be reached for follow-up interviews, it is difficult to know the full range of barriers to care that they experienced. Given that these participants are known to have been distressed, it is also plausible that unresolved symptoms served as barriers to care. Furthermore, some participants suggested that stigma associated with seeking mental healthcare may have represented a barrier to some women. This is a widely recognised barrier and has been noted in several studies.^40,41,42^ Programmes aimed specifically at reducing stigma and increasing awareness amongst peripartum women may be important contributions to maximise the success of future interventions.

For participants, the intervention’s acceptability seemed to lie primarily in the opportunity to talk confidentially to a non-judgemental and empathic person about their problems. Whilst the PST model seems to have had an influence on many participants’ thinking, this appears to have been a secondary benefit for some. This is consistent with evidence from studies to show that task-sharing PST interventions are generally acceptable and feasible to intervention participants in primary care.^22^ However, these findings seem to point to the primary importance of a trusting relationship with an empathic counsellor. Given that task-sharing studies often prioritise intervention models over counsellor skills and qualities, this may have significant implications for future research as well as for practice.

For stakeholders, the programme was generally perceived as expanding and improving the quality of services provided by the facility. Having a professional resource to refer to seemed to relieve them of the pressures of managing distressed patients during the course of routine care. Mental health problems appeared to add to the burden of care experienced by MOU staff who reported not having the time, capacity or skills to manage psychologically distressed patients. In this respect, the intervention was widely deemed to be acceptable by stakeholders. To our knowledge, other studies have not found this. However, the overburdened state of primary healthcare systems might in and of itself represent a barrier to the successful integration of programmes that rely on staff members’ participation. Similar South African studies have shown that stakeholders experience the inclusion of new interventions into usual care as generating additional burden, and that staff buy-in is central to the success of programmes.^20,43^ These findings have important implications for practice and policy. Developing interventions that staff members experience is helpful to their work and not burdensome is likely to be essential to the sustainability of mental health programmes that are integrated into primary care.

There are some limitations of this study. The main limitations are the small sample size and the absence of a control group, restricting our ability to comment on the effect of the intervention. Furthermore, it is possible that participants might have experienced spontaneous remission of symptoms and the study’s positive outcomes simply reflect that. However, these findings suggest that a scaled-up randomised controlled trial of a task-sharing PST intervention to reduce psychological distress amongst pregnant women might have positive outcomes. In addition, both women who participated in the intervention and stakeholders involved in its delivery generally found value in the programme.

## Conclusion

Despite the study’s limitations, in combination with the qualitative data, the outcome data from this study support the feasibility and acceptability of this task shifting brief intervention as well as its potential to effect positive outcomes in the treatment of prepartum psychological distress and CMD symptoms. Perhaps most significantly, the results of this study suggest that integrating mental healthcare interventions into primary care services may improve the mental health of services users, in addition to reducing the burden that patient’s psychological distress may represent for healthcare providers.

## Footnotes

i The authors recognise the deeply and historically problematic nature of this; however, the capacity to monitor developments in health and socio-economic disparities, which originated from such a classification system, is made possible by the continued use of these markers in South Africa

ii The term ‘participants’ is used to denote women who received the PST intervention, whilst the term ‘stakeholders’ represents staff who were involved in the delivery of the intervention.
